# Epigenetic Modifiers in Myeloid Malignancies: The Role of Histone Deacetylase Inhibitors

**DOI:** 10.3390/ijms19103091

**Published:** 2018-10-09

**Authors:** Johanna S. Ungerstedt

**Affiliations:** Department of Medicine, Huddinge, Karolinska Institutet, and Hematology Center, and Karolinska University Hospital, S-141 86 Stockholm, Sweden; johanna.ungerstedt@ki.se; Tel.: +46-8-5858-0000, Fax: +46-8-5858-2525

**Keywords:** myelodysplastic syndromes, acute myeloid leukemia, chronic myelomonocytic leukemia, systemic mastocytosis, treatment, myeloid mutations

## Abstract

Myeloid hematological malignancies are clonal bone marrow neoplasms, comprising of acute myeloid leukemia (AML), the myelodysplastic syndromes (MDS), chronic myelomonocytic leukemia (CMML), the myeloproliferative neoplasms (MPN) and systemic mastocytosis (SM). The field of epigenetic regulation of normal and malignant hematopoiesis is rapidly growing. In recent years, heterozygous somatic mutations in genes encoding epigenetic regulators have been found in all subtypes of myeloid malignancies, supporting the rationale for treatment with epigenetic modifiers. Histone deacetylase inhibitors (HDACi) are epigenetic modifiers that, in vitro, have been shown to induce growth arrest, apoptotic or autophagic cell death, and terminal differentiation of myeloid tumor cells. These effects were observed both at the bulk tumor level and in the most immature CD34^+^38^−^ cell compartments containing the leukemic stem cells. Thus, there is a strong rationale supporting HDACi therapy in myeloid malignancies. However, despite initial promising results in phase I trials, HDACi in monotherapy as well as in combination with other drugs, have failed to improve responses or survival. This review provides an overview of the rationale for HDACi in myeloid malignancies, clinical results and speculations on why clinical trials have thus far not met the expectations, and how this may be improved in the future.

## 1. Introduction to Myeloid Hematological Diseases and Their Treatment

The myelodysplastic syndromes (MDS) are a heterogenous group of clonal myeloid hematological diseases, with a median age of onset of 70 years and a survival of 0.5–8 years, and a 30% risk of transformation to acute myeloid leukemia (AML) [1]. MDS is curable only by allogeneic stem cell transplant, for which only a minority of patients are eligible. The only approved therapy for higher risk MDS is treatment with hypomethylating agents, where two drugs are available, azacitidine and decitabine. Approximately 50–60% of patients respond to therapy [2]. Azacitidine and decitabine are considered to be DNA demethylating agents, as preclinical studies in cell lines have shown a quick and profound global DNA demethylation, as well as site specific promoter demethylation of e.g., P15/INK4B [3,4,5]. However, in vivo and ex vivo studies of MDS patient CD34^+^ progenitor cells do not show a clear DNA demethylation in response to azacitidine, and thus the in vivo mechanism of action of the drugs remains unclear [6,7].

The Philadelphia chromosome negative myeloproliferative neoplasms (MPN) comprises of polycythemia vera (PV), essential thrombocytemia (ET), and myelofibrosis (MF) [1]. The rare diseases chronic neutrophil leukemia and chronic eosinophil leukemia will not be discussed further in this review, nor will the Philadelphia chromosome positive chronic myeloid leukemia (CML). The, *Janus kinase 2* (*JAK2*) V617F mutation occurs in around 95% of PV, and 50% of ET and MF, causing auto-phosphorylation of cytokine receptors and increased JAK-STAT pathway activation [8]. Although the MPNs share a common driver mutation, there are major clinical differences as ET has a largely normal life expectancy, PV has a long life expectancy, whereas MF has a significantly shortened life expectancy [9]. In higher risk disease, oral cytoreductive treatment hydroxycarbamide is used, and for MF, and now also for PV, the JAK2 inhibitor Ruxolitinib may be applied. Allogeneic stem cell transplant may be an option for younger, high risk MF patients [10].

Chronic myelomonocytic leukemia (CMML) is the largest group of MDS/MPN overlap syndromes [1], where proliferative CMML disease is treated like MPN and dysplastic CMML is treated like MDS [11], however responses to azacitidine or decitabine are never long lasting, and there is an imminent need for new treatment options in CMML.

Systemic mastocytosis (SM) is a rare myeloid malignancy [1]. Over 90% of patients carry the D816V activating point mutation in the *KIT* gene. There are two clinical phenotypes, indolent SM with a normal life expectancy and aggressive SM with a poor prognosis [12]. In the aggressive SM group, several therapies have been tested, most recently the pan tyrosine kinase inhibitor Midostaurin [12,13], however to date, no therapy except allogeneic stem cell transplantation has been shown to improve survival, and as the median age at disease onset is around age 70, only a handful of patients are eligible for transplant [14].

Overall, 30% of MDS and CMML patients progress to acute myeloid leukemia (AML). AML is defined by over 20% myeloblasts in the bone marrow, and may be primary (around 75% of all AML cases), secondary (to e.g., MDS, MPN, around 15% of cases) or treatment related e.g., after chemotherapy of hematological or other tumors (10% of cases). Subtypes of AML are well defined according to cell of origin, specific cytogenetic or other aberrations outlined in the WHO diagnostic criteria [1]. In general, patients that are not elderly and unfit, are given intensive chemotherapy (including cytarabine and anthracyclines) to achieve complete remission, and thereafter chemotherapy consolidation or consolidation with allogeneic stem cell transplant in case of high risk disease [15]. However, a large portion of patients are elderly and fragile, and for these patients lower intensity treatment, often similar to regimens given to MDS-patients, can be considered if the AML is not highly proliferative [16]. The 5-year overall survival in adult AML is around 20%.

## 2. Epigenetic Regulation of Normal and Malignant Hematopoiesis

**Figure 1:**
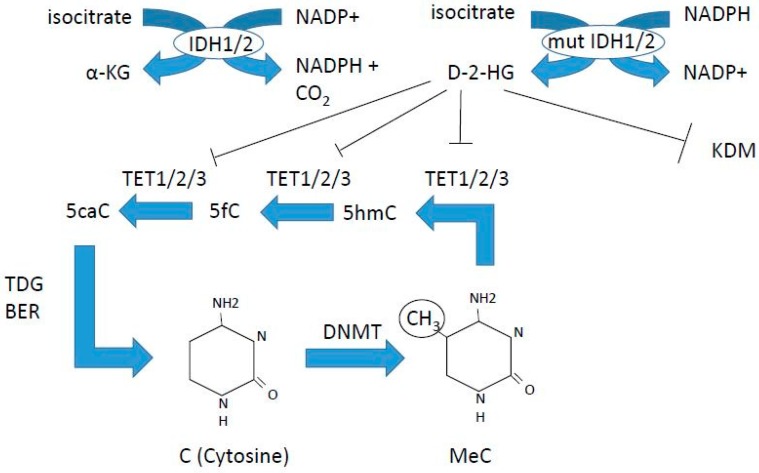
DNA methylation and demethylation. DNMT3A is commonly mutated in acute myeloid leukemia (AML), IDH1, 2 mutations are found in AML, and TET2 is frequently mutated in all myeloid malignancies. Azacitidine and decitabine are DNA demethylating agents, inhibiting DNA methyl transferases (DNMTs).

**Figure 2:**
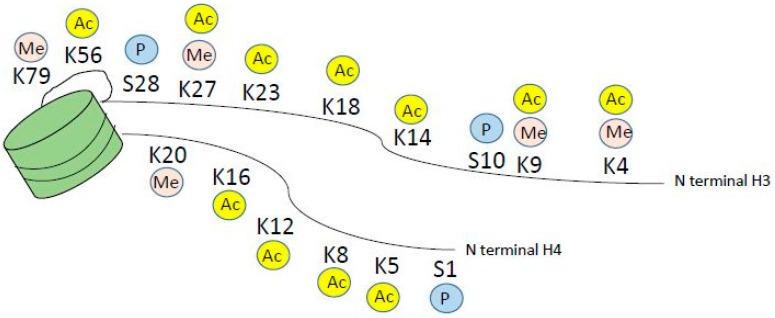
Histone modifications on the N terminal tail of histone H3 and H4. For simplicity, only methylation, acetylation and phosphorylation are depicted, however modifications also include arginine methylation and ubiquitination marks.

Our understanding of how epigenetic regulation of hematopoiesis is orchestrated is rapidly growing. Epigenetics include DNA methylation (Figure 1) as well as covalent, reversible histone modifications (Figure 2) [17,18]. DNA methylation is associated with transcriptional repression via formation of heterochromatin. This is achieved by methylation of the 5-cytosine by DNA methyl transferase (DNMT) enzymes, where maintenance methylation is exerted by DNMT1, and de novo methylation is exerted by DNMT3A and 3B (Figure 1). The gene encoding the DNMT3A enzyme is commonly mutated in AML, leading to loss of function (Table 1). DNA demethylation is a multistep process exerted by the TET enzymes that oxidize 5-methyl cytosine to cytosine (Figure 1). This oxidation process requires α-keto-glutarate, which is produced from isocitrate by isocitrate dehydrogenases 1 and 2 (IDH1 and 2) (Figure 1). Mutations in *IDH1* and *2* are common in AML, and *TET2* mutations are common in all myeloid malignancies (Table 1). Table 1 summarizes the currently known mutations in epigenetic regulators found in myeloid malignancies, and is compiled from the pivotal studies of Papaemmanuil [19] and Haferlach [20] for MDS, and Ley for AML [21], as well as reviews for CMML [22], MPN [23], SM [24], and references [17,18,25,26] for comparison of mutation frequencies reported.

**Figure 3:**
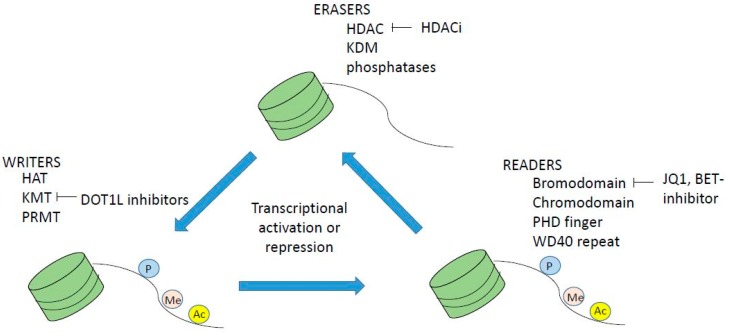
Epigenetic writers are histone acetyl transferases HAT (KAT), histone lysine methyl transferase (KMT) and PRMTs (protein arginine methyl transferases), readers are bromodomain proteins like BET family proteins, and erasers are histone deacetylase inhibitors (HDACi), KDM (lysine/histone demethylases) and phosphatases. Inhibitors or writers, readers and erasers are being developed and are in clinical trials for myeloid malignancies, for example HDACi, bromodomain BET inhibitor Q1 and DOT1L inhibitors, of which the latter are in clinical phase I trials.

Histone modifications occur on the n-terminal protruding tail of predominantly histone H3 and H4, and consist of lysine residues being acetylated or methylated, and serine residues can be phosphorylated (Figure 2). In addition, arginine may be methylated however this will not be further discussed as it is outside the scope of this paper. Histone acetylation occurs via a family of lysine acetyl transferases (KAT), formerly called histone acetyl transferases (HAT), and histone lysine methyl transferases (KMT) methylate histones. KAT and KMT enzymes along with protein arginine methyl transferases are called epigenetic writers, and HDAC, histone lysine demethylases (KDM) and phosphatases are called epigenetic erasers (Figure 3), reviewed in reference [27]. In addition, there are readers that read the epigenetic code. These are bromodomain containing proteins like the BET family, chromodomain, PHD finger and WD 40 repeats (Figure 3). Inhibitors to both writers, e.g., DOT1L [28,29] and readers e.g., BET inhibitor JQ1 [30] are in development and early clinical trials for myeloid malignancies

## 3. Dysregulation of Histone Acetylation and Methylation in Myeloid Malignancies

**Figure 4:**
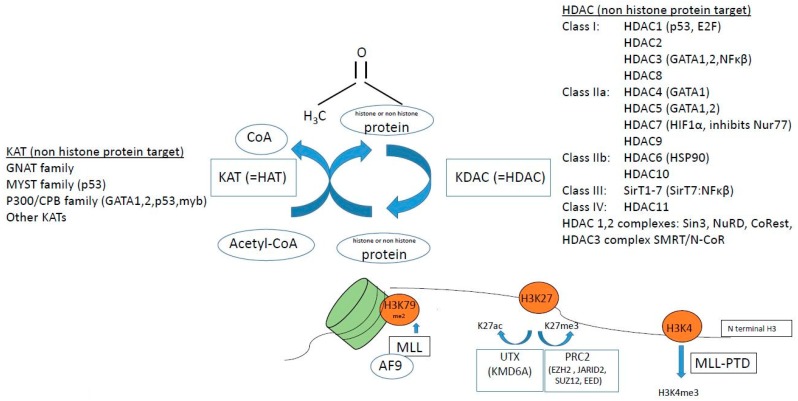
Factors associated with histone or non-histone protein lysine acetylation/methylation, affected in hematological malignancies. In brackets are listed non-histone targets of HAT (KAT) and histone deacetylases (HDACs). See text for details on how these epigenetic factors are associated with myeloid malignancies.

KATs modulate the process of hematopoiesis both via altering the epigenetic status of chromatin via histone lysine acetylation [18], and via regulation of non-histone protein acetylation [31]. Mutations in KATs, e.g., *CBP* and *p300* have been described in myeloid malignancies, although they are rare events (Table 1, Figure 4). KAT6A (MOZ/myst3, part of the MYST family of KATs) is important in regulating hematopoietic stem cells, and is a target of translocations causing AML. Recently, Baell et al. elegantly showed that KAT6A/B (MOZ/myst 3 and myst 4) inhibitors arrest tumor growth and induce senescence in AML cells, in vitro and in vivo [32]. UTX (KDM6A) acetylates H3K27ac thus mediating active chromatin, and *UTX* mutations are found in AML (Table 1) [33].

In AML subtype MLL-PTD (MLL mixed lineage leukemia), a mutation causes a partial tandem duplication (PTD) that confers excessive tri-methylation of H3K4 (Figure 4). MLL, which is a KMT, may also have over 50 different translocation partners, one of which is MLL-AF9 that recruits DOT1L to methylate H3K79me2 (Figure 4). The *myb* oncogene requires myb–p300 interaction for leukemic transformation of AML oncogenes AML-ETO and MLL-AF9 [34], thus there are many implications of histone methylation in leukemia.

Mutations in the *TP53* gene encoding the P53 tumor suppressor occurs in approximately 10% of AML and MDS patients, and is associated with a dismal prognosis. The TP53 gene requires coactivator CBP/p300 acetylation for full transcriptional activation, where the KAT p300 acetylates p53. P53 is normally de-acetylated by HDAC1. In addition, in AML with inv(16) or t(16;16), p53 activity is inhibited via interactions between the inv(16) fusion protein CBFβ-SMMHC with HDAC8, where HDAC8 aberrantly deacetylates p53, which promotes leukemia. Inhibition of HDAC8 restores p53 and induces apoptosis selectively in leukemic inv(16)^+^ CD34^+^ cells but spares normal CD34^+^cells [35].

The HDAC3 containing NCoR complex can be recruited by the oncogenic fusion proteins AML-ETO and PML-RARα, and HDAC1 knockdown increases survival in PML-RARα mediated APL. To add to the complexity of HDAC function, a specific HDAC may have different roles over time in leukemia development. The most well studied example is acute promyelocytic leukemia (APL), where a translocation t(15;17) generates the PML-RARα fusion protein. Normally, RAR (retinoic acid receptor) is a transcription factor and when retinoic acid is absent, RAR associates with HDAC1/2 containing complex SMRT/N-CoR and represses transcription. Retinoic acid causes release of the corepressor complex and leads to transcription. In APL cells, retinoic acid does not release the corepressor complex, resulting in a differentiation block. However, pharmacological doses of retinoic acid, as used in the clinic, degrade the fusion protein and the cells differentiate. Thus, HDAC play an important role in APL, however their role varies over time as *HDAC1/2* knockdown in early leukemogenesis expands the leukemia, whereas in leukemic phase, knockdown of the same HDAC1/2 cause differentiation and apoptosis of APL cells, and increased survival of APL mice [36]. The polycomb repressive complex 2 (PRC2) silences H3K27 to H3K27me3, and several genes encoding components of the PRC2; *EZH2*, *SUZ12*, *JARID2* and also mutations in *ASXL1* coding for the PRC2 associated protein ASXL1, have been found to be mutated in myeloid malignancies (Table 1, Figure 4) [17,18,19,20,21,22,23,25,26].

HDAC enzymes (KDACs) are commonly mutated in solid tumors, with 30% of endometrial tumors having HDAC mutations, however only 2% of patients with AML have HDAC mutations [36]. A thorough investigation of HDAC gene expression in MDS and AML showed that in myeloid malignancies, HDAC expression is heterogenous, with no clear pattern of over- or under-expression of any HDAC [37]. However, in the CD34^+^ progenitor compartment of patients with MF, there is an increase in HDAC levels [38]. Interestingly, HDACs actually preceded the histone proteins phylogenetically, clearly indicating that HDACs primarily target non-histone protein substrates [39], and many of these non-histone proteins are the products of tumor suppressor genes, oncogenes or transcription factors important for hematopoiesis (Figure 4) [31], and as they are deacetylated by HDACs, they are targets of HDACi treatment.

## 4. Preclinical Experience of HDACi in Myeloid Malignancies

In general, HDACi treatment alters the expression of 5–10% of transcribed genes, depending on cell type. The specific antitumor activity of HDACi varies between tumor types, and the antitumor activity on a specific tumor type may vary between different HDACi. The general mechanisms for HDACi induced cell death include apoptosis or autophagy, increasing ROS production and decreasing scavengers, increasing DNA damage and decreasing DNA repair, decreasing oncoprotein expression and stability and stimulating immunogenic cell death [27,40,41]. In myeloid malignancies, HDACi treatment induces cell death, growth arrest or differentiation, activating chromosome degradation, altering angiogenesis, inactivating chaperone complexes, and inducing expression of cell cycle inhibitors e.g., p21, and of pro-apoptotic genes [42,43,44,45,46,47]. The DNA damage induced by HDACi can be repaired by normal cells but not by transformed cells [48].

Induction of apoptosis is the major route for HDACi induced cell death, and it may be via either the intrinsic (mitochondria) or extrinsic (death receptor) pathway. The specific changes in gene expression leading to apoptosis in myeloid malignancies varies between the different HDACi, but frequently, extrinsic pathway via TRAIL is used. In AML, HDACi MS275 treatment induces the expression of *TRAIL* by activating the *TNFS10* gene that encodes TRAIL, triggering death signal via the extrinsic pathway, and additionally RNA interference against *TRAIL* blocked downstream caspase activation and inhibited MS275 mediated apoptosis, suggesting that at least in AML cells, MS275 mechanism of action is via TRAIL [44]. In K562 leukemic cells, VPA reduces the expression of *c-FLP* and *Bcl.2/Bcl-xL* anti-apoptotic factors, as well as sensitized cells to TRAIL/Apo2L mediated apoptosis, thus acting on both the intrinsic and extrinsic apoptotic pathways [49]. In APL and AML1-ETO mouse models in vivo and human cell lines in vitro, HDACi valproic acid upregulates *TRAIL*, *DR5*, *FasL* and *Fas* in leukemic cells but not normal progenitors, thus for the sensitivity of HDACi to leukemia a transformed phenotype is required [42].

In a recent study of several AML cell lines as well as CD33^+^ progenitor cells from AML and MDS patients, vorinostat induced gene expressions of *COX2*, *p15*, *cFOS*, genes that are downregulated in MDS and AML, and suppressed overexpressed genes *cyclin D1* and *c-MYC* [50]. This led to cell cycle arrest, terminal differentiation and or apoptosis, via mechanisms including modulation of SP1 [50]. Recently, HDACi entinostat has been shown to restore the decreased orphan nuclear receptor *Nur77* expression in AML cell lines and in AML patient leukemia cells, especially in the leukemic stem/very early CD34^+^/38^−^ progenitors, and induce apoptosis, presenting a novel mechanism of action of HDACi and suggesting that Nur77 may be a biomarker for HDACi apoptotic effect [51]. Thus, HDACi have multiple and broad effects, and likely the mechanism of HDACi induced tumor cell death may be depending on the molecular defects of the target cell, as well as of the specific HDACi used [43].

In AML/ETO, single agent valproic acid inhibits not only the mature leukemic cells but also immature progenitors by targeting the AML1/ETO-HDAC complex SMRT/N-CoR, inducing differentiation [52]. In MPN, preclinical data strongly supports the effect of HDACi inhibiting proliferation and inducing apoptosis in *JAK2* mutated cells, normalizing splenomegaly and blood counts in JAK2 mutant knock-in mice [53] and promoting proteasome mediated JAK2 degradation by disrupting HSP90 chaperone function. Treatment of JAK2 mutated CD34^+^ progenitor cells with panobinostat induces apoptosis and inhibits JAK2 expression and activity, subsequently reducing pSTAT3, pSTAT5, pAKT and pGATA1, and partially inhibiting the binding between HSP90 and JAK2, suggesting that acetylation of HSP90 could mediate JAK2 degradation [54]. The same study showed a synergistic effect of addition of JAK2 inhibitor to panobinostat.

HDACi therapy in SM was first assessed in a canine model of SM [55]. Our group has shown that several first and second generation HDACi dose dependently inhibit growth and induce apoptosis in KIT D816V mutated SM cell lines, and that vorinostat selectively kills *KIT D816V* mutated primary patient mast cells whereas normal mast cells are unaffected [56]. To support epigenetics in the pathogenesis of SM, a recent study shows a deficiency of lysine methylation in aggressive SM [57]. However, to date there have been no clinical trials of HDACi in SM.

## 5. Preclinical Rationale for Combination Therapy Including HDACi

There is a large body of preclinical evidence showing synergistic effects of various first and second generation HDACi in combination with azacitidine or decitabine in MDS and AML cell lines or ex vivo cultured patient cells. These include enhanced growth arrest, inhibition of DNA synthesis and loss of clonogenic potential, and synergistic effects in re-expressing silenced genes [58,59,60]. When combining panobinostat and decitabine in vitro, there were synergistic effects in attenuating DNMT1 and EZH2, de-repression of JunB and enhanced leukemic cell death [61]. Primary AML patient CD34^+^ cells were more sensitive than normal CD34^+^ cells to the treatment, indicating a specific anti-leukemic effect and that normal progenitors are spared. In a molecular study of clinical samples from the clinical trial of Tan et al., using azacitidine and panobinostat for MDS and AML [62], Liu et al. analyzed mRNA of *Nur77*, *p15* and *p21* in the clinical patient samples, and found that restored levels of *Nur77* and *p21* correlated with clinical responses to the combination therapy [59], in concordance with other studies suggesting *Nur77* as a biomarker of HDACi mediated apoptosis also in the leukemic stem cell compartment [51]. In MPN, HDACi have been shown to synergize with JAK2 inhibitors in inducing apoptosis in JAK2 mutated cells [54].

## 6. Results from Clinical Studies of HDACi Monotherapy and Combination Therapy for Myeloid Malignancies

Single agent first and second generation HDACi have been tested in several small phase I and II studies for MDS and AML, showing low overall response rates and 0–10% partial or complete remissions, reviewed by Morabito et al. and by Stahl et al. [63,64], with the conclusion that combination treatment is needed to achieve a clinical effect. However, despite preclinical support for synergistic effects of combination therapy since the pivotal study of Cameron et al. in 1999, showing synergy of demethylaton and HDAC inhibition in re-expressing silenced genes in cancer [58], and several studies since references [59,60,61,62], and early phase I studies showed promising results, both for vorinostat in combination with decitabine [65] and panobinostat in combination with azacitidine [62,66], thus far, the randomized phase II clinical trials of various doses and various HDACi drugs in combination with azacitidine or decitabine for MDS, CMML and AML have not been able to show an improved clinical outcome. The recent phase II studies in MDS, CMML and AML are summarized in Table 2. In addition, a meta-analysis of these trials has been recently published [67]. Of note, there are currently 156 clinical trials of HDACi mono- or combination therapy registered at clinicaltrials.gov, using a plethora of HDACi agents (Table 3). 

A review of valproic acid effects on AML cells conclude that single agent valproic acid may stabilize disease in the many old and fragile AML patients that are unfit for more intensive therapy, however as of yet, no randomized studies have been conducted [75]. However, there is an ongoing prospective randomized multicenter phase II trial of low dose decitabine alone or in combination with valproic acid and all-trans-retinoic acid in patients with AML, ineligible for induction chemotherapy, is also ongoing and an interim report has been published [76]. Thus, there may be therapeutic options of epigenetic drugs also for the elderly, fragile patients that are not eligible for more intense therapy.

A number of HDACi have been investigated in clinical trials in MPN, recently reviewed by Bose and Verstovsek [77]. Overall, HDACi monotherapy is effective in MPN however not well tolerated in ET and PV patients, where published studies show significant drop out due to toxicity, even if HDACi monotherapy clearly is active and also decreases JAK2 mutation burden in PV and ET [78,79,80]. Some studies have reported toxicity and high dropout rate also in MF [81], however a recent follow up study on panobinostat monotherapy in primary MF and post PV/ET MF showed a response rate of 36% according to IWG-MRT criteria, with a median spleen volume reduction of 34% in eight evaluable patients, of which one obtained a complete molecular response and six patients remained on therapy for a median of 18 months [82]. Bose and Verstovsek conclude that that the combination of HDACi with JAK2 inhibitor in MF is the most promising approach, however toxicity and long-term tolerability may be future concerns [77]. Currently, three clinical phase I/II trials using HDACi in combination with JAK2 inhibitor ruxolitinib are ongoing, NCT01693601 (the Prime study, panobinostat and ruxolitinib, likely to end Feb 2019), NCT01433445 with panobinostat and ruxolitinib, currently in expansion phase, and NCT02267278 with pracinostat and ruxolitinib). Overall, preclinical data for combination therapy in MF is solid and there are great expectations on the ongoing combination trials of HDACi and ruxolitinib in MF. For SM, there have been no clinical trials including HDACi therapy until now.

## 7. Why Have the Clinical Studies Failed?

Four recent reviews on the combination trials of azacitidine or decitabine with HDACi conclude that there may be still a future for the drug combination, despite the lack of beneficial results in phase II trials [64,83,84,85]. As preclinical data on how to best combine the drugs is lacking, it may well be that the clinical trials have administered the drugs with suboptimal timing. Simultaneous administration with varying dose intervals has been used, perhaps inducing pharmacological antagonism as azacitidine requires cell division and DNA replication to exert its effects, and HDACi inhibit cell division and proliferation, thus potentially antagonizing the effect of azacitidine. Another issue that must be met is how to choose the optimal HDACi for the specific target patient population, regarding selectivity of inhibition of target proteins, and regarding the pattern of somatic mutations and chromosomal abnormalities of each patient. Here, novel more selective HDACi are being developed, with focused targets. In addition, the mechanism of action of HDACi in MDS and AML is unclear. In fact, despite azacitidine and decitabine being widely used for over 10 years, the mechanism of action of these drugs in vivo is still unknown, and we, as well as others, have failed to demonstrate demethylation of MDS progenitor cells upon azacitidine treatment [6,7]. In addition, there are to date no established biomarkers to assess azacitidine or decitabine effects, nor are there any established biomarkers for monitoring HDACi effects. Thus, we have no readout for either of the drugs and thus no means of elucidating which drug is failing, when we combine them in the clinical setting. Currently our only readout is remission and survival, and possibly decrease of a mutated clone size, however we cannot measure if the drug effects are counteracting each other as we have no biomarkers of treatment effect. Thus, before attempting new clinical trials, we need to solve the issue of how to combine the drugs to optimize synergy and decrease the risk of antagonism or inhibition, and in addition we imperatively need to establish reliable biomarkers of drug effects.

## 8. Summary

Despite a theoretical rationale and profound preclinical proof of HDACi efficacy in myeloid malignancies, all phase II randomized clinical trials have failed, except for the combination of HDACi with JAK2 inhibitor ruxolitinib in MF. However, optimizing combination treatment strategy, e.g., sequential treatment and not simultaneous, will be a key issue to avoid pharmacological antagonism, and requires further basic in vitro studies of optimizing drug scheduling and doses, as well as biomarkers to follow in vivo drug effects. In addition, novel, more selective HDACi should be preferred, avoiding off target effects. In conclusion, there may still be a role for HDACi in myeloid malignancies, beyond the promising combination therapy of HDACi and ruxolitinib for MF.

I apologize to all authors that have made significant contributions to the field but was not cited in the current review, due to practical space limitations.

## Figures and Tables

**Table 1:** In all types of myeloid malignancies, genetic alterations in epigenetic modifiers are found, however the mutation frequency varies between diseases. For references please see text.

Function	Gene	Loss/Gain of Function	Activity	Frequency in Myeloid Malignancies
DNA methylation	*DNMT3A*	loss	De novo DNA methylation	AML 12–22%
				MDS 5–10%
				CMML 5%
				MPN 7–15%
				ASM 1%
DNA methylation	*TET2*	loss	5-methyl-C to 5-hydroxy methyl-C	AML 7–23%
				MDS 20–25%
				CMML 60%
				MPN 4–13%
				ASM 40%
DNA methylation	*IDH1/2*	gain	Cofactor for TET2	AML 10–30%
				MDS 3%
				CMML 1–10%
				MPN 2.5–5%
Histone methylation	*EZH2*	Loss	Trimethylation of H3K27, part of PRC2 complex	AML rare
				MDS 6%
				CMML 5%
				MPN 3–13%
				ASM 3%
Histone methylation	*ASXL1*	loss	Associates with PRC1 and PRC2	AML 5%
				MDS 15–20%
				CMML 40–45%
				MPN 2–23%
				ASM 14%
Histone methylation	*SUZ12*	loss	Member of PRC2	MDS rare, <1%
Histone methylation	*EED*	loss	Member of PRC2	MDS rare, <1%
Histone methylation	*KMT2A* (*MLL1*)	gain	H3K4 lysine methyl transferase	AML 5%
				MDS/AML 5%
Histone methylation	*MECOM* (*EVI1*)	gain	H3K9(me1) lysine methyl transferase	MDS/AML rare
Histone methylation	*PRDM16*	gain	H3K9(me1) lysine methyl transferase	MDS/AML rare
Histone methylation	*SETD2*	loss	H3K36 lysine methyl transferase	AML 5%
Histone methylation	*JARID2*		Recruits PRC2 to target	sAML(from MDS, MPN) 6.5%
				MDS, MPN 0.2%
Histone methylation	*UTX* (=*KDM6A*)	loss	Counteracts PRC2 by removing di and trimethylated H3K27	AML 3%
				MDS 2.5%
				CMML 8%
				MDS/MPN 4.8%
Histone acetylation	*CREBBP* (*CBP*)	gain	Lysine acetyl transferase	AML rare
Histone acetylation	*P300* (*EP300*)	gain	Lysine acetyl transferase	AML rare
Histone deacetylation	*HDAC2*	loss	Lysine deacetylase	AML rare
Histone deacetylation	*HDAC3*	loss	Lysine deacetylase	AML rare

**Table 2:** Phase I/II trials with combination treatment of HDACi and hypomethylating agents, in AML and MDS, sometimes including CMML. OS = overall survival, ORR = overall response rate, CR = complete remission. In the studies by Uy et al., and Tan et al., there was no control arm thus a comparison of efficacy to monotherapy could not be made. Out of five evaluable studies, none showed an advantage of combination therapy. ^1^ Azacitidine 75 mg/m^2^ Day 1–5/28, ^2^ panobinostat 3 days/w 7 doses/28 days, phase II 30 mg oral daily Day 1–7/28, ^3^ decitabine 20 mg/m^2^ iv Day 1–5, ^4^ valproic acid 50 mg/kg oral Day 1–7/28, ^5^ azacitidine 75 mg/m^2^ Day 1–7/28, ^6^ vorinostat 300 mg twice daily Day 3–9/28, ^7^ panobinostat 20–40 mg Day 3, 5, 8, 10, 12, 15, in phase IIb 40 mg, ^8^ pracinostat 60 mg or placebo oral every 2 days Day 1–21/28, ^9^ azacitidine 50 mg/m^2^ 10 days, ^10^ entinostt 4 mg/m^2^ Day 3, 10/28, ^11^ panobinostat three times/week during two weeks/4, phase I dose escalation to 50 mg, phase II 40 mg.

Study, Trial Number and Reference	Disease, Phase	Additive Clinical Effect of HDACi	Drugs	Clinical Response	Molecular Markers Analyzed
Tan [62], ACTRN12610000924055, Open label, phase Ib/II	Higher risk MDS, AML. *n* = 39	NA	Azacitidine ^1^, Panobinostat ^2^	ORR 31% in AML, 50% in MDS. Median OS 8 months in AML, 16 months in MDS.	Total PBMC histone H3 and H4 acetylation higher in responders. NUR77 and p21 markers of treatment efficacy [59]
Issa [68], NCT00414310, Randomized, Phase II	Higher risk MDS, AML. *n* = 149	NO	Decitabine ^3^, valproic acid ^4^	No improvement in CR or OS with adding valproic acid.	NO
Sekeres [69], NCT01522976, Randomized, Phase II	Higher risk MDS, CMML. *n* = 184	NO	Azacitidine ^5^, Vorinostat ^6^	ORR 38% monotherapy, 27% combination (*p* = 0.16). Study not powered for calculating OS.	NGS. ORR was higher in DNMT3A mutated patients. ORR lower for SRSF2 and ASXL1. Response duration low in TET2 and TP53 mutated patients.
Garcia-Manero [70], NCT00946647, Randomized phase Ib/II	MDS, CMML AML with 20–30% blasts. *n* = 113	NO	Panobinostat ^7^, Azacitidine ^5^	CR 27.5% in the combination arm, 14.3% in monotherapy. No difference in OS or time to progression.	NGS data on 24 myeloid mutations, no clear correlation between mutation pattern and response.
Garcia-Manero [71], NCT01873703, Randomized phase II, double blinded	MDS (up to 30% blasts). *n* = 102	NO	Azacitidine ^5^, Pracinostat ^8^	CR 18% in the combination group, 33% in monotherapy group (*p* = 0.07). No difference in OS (16 vs. 19 months).	NO
Prebet [72], NCT00313586, Prebet [73], Open label phase II	MDS, CMML, MDS/AML. *n* = 149	NO	Azacitidine ^9^, entinostat ^10^	OS 18 months for monotherapy, 13 for combination.	No correlation between overall methylation decrease and clinical response, or with treatment arm. Possible correlation of SOCS1 methylation and response.
Uy [74], NCT00691938, Open label observational phase I/II	AML, MDS. *n* = 52	NA	Decitabine ^3^, panobinostat ^11^	ORR 11/37 AML and 7/14 MDS, total 36% ORR. Median OS 6.4 months.	Extensive sequencing, complex patterns. Mutations persist during complete remission.

**Table 3:** HCACi that are listed at clinicaltrials.gov, with at least one listed phase I clinical trial.

Drug Type	Compound	Name	Selectivity	Clinical Status	Used in Myeloid Disease
Hydroxamates	MK0653 (SAHA)	Vorinostat	Pan HDACi	Phase II/III. Approved.	Yes. Single and combination
	LBH589	Panobinostat	Pan HDACi	Phase II/III. Approved.	Yes. Single and combination
	PXD101	Belinostat	Pan HDACi	Phase I/ II/III. Approved.	Yes. Combination therapy
	JNJ-26481585	Quisinostat	HDAC1,3,5,8	Phase I/II	MDS and AML. Single therapy
	ITF2357	Givinostat	Class I and II	Phase I/II	MPN. Single and combination
	SB939	Pracinostat	Class I, II, IV	Phase II	Yes. Single and combination
	SHP141	Remetinostat		Phase II/III	No
	4SC201	Resminostat	Pan HDACi	Phase I/II	No
	4SC202	Domatinostat	HDAC1,2,3	Phase I/II Approved in melanoma (combination)	Yes. Single therapy
	ACY1215	Ricolinostat	HDAC6	Phase I/II	No
Cyclic tetrapeptides	FK228	Romidepsin	Class I	Phase I/II/III. Approved.	Yes. Single and combination
Benzamides	MS275	Entinostat	HDAC1,2,3	Phase I/II	Yes Combination therapy
	MGCD0103	Mocetinostat	Class I	Phase I/II	Yes Single and combination
Fatty acids	Valproic acid	Valproate	Class I and IIa	Phase I/ II	Yes Combination therapy
	Sodium Butyrate	Butyrate	Class I and IIa	Phase I/II	Mostly non cancer diseases
